# IGF-1 Restores Visual Cortex Plasticity in Adult Life by Reducing Local GABA Levels

**DOI:** 10.1155/2012/250421

**Published:** 2012-06-06

**Authors:** José Fernando Maya-Vetencourt, Laura Baroncelli, Alessandro Viegi, Ettore Tiraboschi, Eero Castren, Antonino Cattaneo, Lamberto Maffei

**Affiliations:** ^1^Laboratory of Neurobiology, Scuola Normale Superiore, Piazza dei Cavalieri 7, 56100 Pisa, Italy; ^2^Institute of Neuroscience, CNR, Via Moruzzi 1, 56100 Pisa, Italy; ^3^Neuroscience Centre, University of Helsinki, 00014 Helsinki, Finland

## Abstract

The central nervous system architecture is markedly modified by sensory experience during early life, but a decline of plasticity occurs with age. Recent studies have challenged this dogma providing evidence that both pharmacological treatments and paradigms based on the manipulation of environmental stimulation levels can be successfully employed as strategies for enhancing plasticity in the adult nervous system. Insulin-like growth factor 1 (IGF-1) is a peptide implicated in prenatal and postnatal phases of brain development such as neurogenesis, neuronal differentiation, synaptogenesis, and experience-dependent plasticity. Here, using the visual system as a paradigmatic model, we report that IGF-1 reactivates neural plasticity in the adult brain. Exogenous administration of IGF-1 in the adult visual cortex, indeed, restores the susceptibility of cortical neurons to monocular deprivation and promotes the recovery of normal visual functions in adult amblyopic animals. These effects were accompanied by a marked reduction of intracortical GABA levels. Moreover, we show that a transitory increase of IGF-1 expression is associated to the plasticity reinstatement induced by environmental enrichment (EE) and that blocking IGF-1 action by means of the IGF-1 receptor antagonist JB1 prevents EE effects on plasticity processes.

## 1. Introduction

The brain processes information from the external world and creates neuronal representations of the environment that change in response to sensory experience [1]. The extent to which environmental stimuli modify brain structure and function has been extensively studied in the visual system. Visual cortical circuitries are highly sensitive to experience during well-defined temporal windows in early life, known as critical periods (CPs), but this phase of heightened plasticity decreases over postnatal development [2, 3]. Pioneering electrophysiological studies demonstrated that occluding one eye early in development (monocular deprivation, MD) leads to an ocular dominance (OD) shift of cortical neurons, that is, a reduction in the number of cortical cells responding to that eye and an increment of neurons driven by the open eye [4, 5]. In addition, the deprived eye becomes amblyopic: its acuity and contrast sensitivity are dramatically reduced. Because MD does not trigger amblyopia in adulthood, this early temporal window characterized by enhanced plasticity in response to experience is a typical example of a CP.

Converging evidence attributes the decline of plasticity that occurs with age to the maturation of intracortical inhibitory circuitries [6–8]. A direct demonstration that GABAergic inhibition is a crucial brake limiting plasticity in the adult visual cortex (VC) derives from a study showing that the pharmacological reduction of intracortical inhibition reactivates OD plasticity in adult life [9]. Accordingly, different experimental approaches that shift the cortical inhibitory/excitatory balance have been reported to trigger the reinstatement of neural plasticity in the adult brain [10–15].

Environmental enrichment (EE) is an experimental paradigm characterized by enhanced sensory-motor and cognitive stimulation that has a profound impact on brain structure and function [16, 17]. It has been recently demonstrated that short periods of EE in adulthood reactivate juvenile-like plasticity in the visual system, promoting both a marked OD shift of cortical neurons in response to MD and the recovery of normal visual functions after long-term sensory deprivation [12, 18, 19], reviewed in [17, 20]. This has prompted the search for endogenous factors with the potential to enhance experience-dependent plasticity (enviromimetics) as a therapeutic strategy for brain repair in adult life.

A candidate molecule that might be exploited to reproduce the beneficial effects of EE is insulin-like growth factor 1 (IGF-1). IGF-1 is a peptide implicated in early phases of brain development, and there is evidence that IGF-1 underlies the effects caused by physical activity in synaptic plasticity and hippocampal neurogenesis [21–24]. It has been reported that MD during the CP increases the expression of IGF-1 binding proteins and affects different genes of the IGF-1 pathway [25]. In addition, exogenous administration of IGF-1 prevents the physiological effect of MD during the CP [25]. Developmental studies using EE as a strategy to assess environmental influences on brain function further confirm the role of IGF-1 in mediating experience-dependent plasticity. In rodents, EE increases IGF-1 cortical levels and accelerates development of normal visual functions, whereas blocking IGF-1 signaling in EE animals prevents this plastic phenomenon [26, 27]. In humans, enriching the environment in terms of body massage accelerates the developmental maturation of visual functions  and, as in the offspring of rats, this phenomenon is accompanied by enhanced IGF-1 levels [28].

Despite these findings, a thorough analysis of IGF-1 effects on adult visual cortical plasticity is still missing. Here, we addressed this issue by using two classical paradigms of experience-dependent plasticity: (i) the shift of OD in response to MD and (ii) the recovery of visual functions in adult amblyopic animals. In the rat, these two plastic events are restricted to the CP during early stages of development. We report that intracortical IGF-1 administration reactivates neural plasticity in the adult VC. These effects were accompanied by a marked reduction of intracortical GABA levels. Moreover, we show that a transitory increase of IGF-1 expression accompanies the reinstatement of plasticity caused by EE in adulthood and that blocking IGF-1 action by means of the IGF-1 receptor antagonist JB1 prevents EE effects on plasticity processes.

## 2. Methods

### 2.1. Subjects

A total of 92 adult Long-Evans hooded rats at the postnatal day 70 (P70) were used in this study, which was approved by the Italian Ministry of Public Health. Animals were group-housed under standard conditions with food and water *ad libitum* in Plexiglas cages (40 × 30 × 20 cm) and kept in a 12 : 12 light/dark cycle.

### 2.2. Surgical Treatments

To assess OD plasticity, MD was performed by eyelid suturing. Animals were anesthetized with avertin (1 mL hg^−1^), mounted on a stereotaxic apparatus, and eyelid closure performed by sewing of the eyelids using sterile-surgical sutures. Eyelid closure was inspected daily until complete cicatrization, and subjects with even minimal spontaneous reopening were excluded. Great care was taken to prevent inflammation or infection in the deprived eye through topical application of antibiotic and cortisone.

To assess amblyopia recovery, young animals (P21) were anesthetized with avertin (1 mL hg^−1^) and monocularly deprived by eyelid suture. Eyelid occlusion was inspected daily until complete cicatrization; subjects with even minimal spontaneous re-opening were excluded. Adult long-term deprived (amblyopic) rats were then subjected to reverse suture (RS) in parallel to the intracortical infusion of IGF-1 or vehicle solution; that is, the long-term deprived eye was opened while the other eye was sutured shut during two weeks. Great care was taken during the first days after RS to prevent inflammation or infection in the previously deprived eye through topical application of antibiotic and cortisone.

### 2.3. Intracortical Administration of IGF-1 and JB1

Adult rats (P70) were anesthetized with avertin (1 mL hg^−1^) and osmotic minipumps, connected via PE tubing to a stainless steel cannula (30 gauge), implanted in the VC for 2 weeks. Osmotic minipumps (14-day flow rate: 0.5 *μ*L hr^−1^) were filled up with IGF-1 (0.5 *μ*g *μ*L^−1^), the IGF-1 receptor antagonist JB1 (10 *μ*g mL^−1^), or vehicle solution (saline) and implanted 4.5 mm lateral and 2 mm anterior to *λ* (i.e., 2 mm distant from the site of electrophysiological recordings) in the VC contralateral to the deprived eye, as previously described [12, 15]. This treatment caused no damage in the binocular region of the VC where electrophysiological recordings were performed.

### 2.4. In Vivo Electrophysiology

After respective treatments, adult rats (P85–90) were anesthetized with urethane (0.7 mL hg^−1^; 20% solution in saline) by i.p. injection and placed in a stereotaxic frame. Additional doses of urethane were used to keep the anesthesia level stable throughout the experiment. Body temperature was continuously monitored and maintained at ~37°C by a thermostated electric blanket during the experiment. An ECG was continuously monitored. A hole was drilled in the skull, corresponding to the binocular portion of the primary VC (Oc1B) contralateral to the deprived eye. After exposure of the brain surface, the dura was removed, and a micropipette (2 MΩ) filled with NaCl (3 M) was inserted into the cortex 5 mm lateral and 0 mm anterior to *λ* (i.e., 2 mm distant from the injection site in which no damage of the cortex was observed). Both eyes were fixed and kept open by means of adjustable metal rings surrounding the external portion of the eye bulb. Alterations of binocularity and visual acuity (VA) were measured by using visual evoked potentials (VEPs). To record VEPs, the electrode was advanced at a depth of 100 or 400 *μ*m within the cortex. At these depths, VEPs had their maximal amplitude [29]. Signals were band-pass-filtered (0.1–100 Hz), amplified, and fed to a computer for analysis, as described previously [7]. Briefly, at least 128 events were averaged in synchrony with the stimulus contrast reversal. Transient VEPs in response to abrupt contrast reversal (0.5 Hz) were evaluated in the time domain by measuring the peak-to-baseline amplitude and peak latency of the major negative component. Visual stimuli were horizontal sinusoidal gratings of different spatial frequencies and contrast, generated by a VSG2/2 card running custom software and presented on a monitor (20 × 22 cm; luminance 15 cd m^−2^) positioned 20 cm from the rat's eyes and centered on the previously determined receptive fields. VA was obtained by extrapolation to zero amplitude of the linear regression through the last four to five data points in a curve where VEP amplitude is plotted against log spatial frequency. Binocularity (OD) was assessed calculating the contralateral to ipsilateral (C/I) VEP ratio, that is, the ratio of VEP amplitudes recorded by stimulating the eye, respectively, contralateral and ipsilateral to the VC where recording is performed. To prevent sampling bias, VEPs were recorded at three different penetrations in Oc1b and at 100 *μ*m and 400 *μ*m depths for each penetration.

### 2.5. In Vivo Brain Microdialysis

To perform brain microdialysis, adult rats (P85–90) were anesthetized and stereotaxically implanted with stainless steel guide shafts above Oc1B at coordinates: 7.3 mm posterior to bregma, 4.4 mm lateral to the midsagittal suture, and 1 mm ventral to the skull, as previously described [11, 12]. One week later, *in vivo* sampling of dialysates was performed inserting a microdialysis probe into the guide shaft previously implanted. The probe was made of concentric fused-silica polyimide-covered capillary tube into a 26-gauge stainless steel tube with a 1 mm long tip of exposed cellulose membrane (6000 MW cutoff). It was connected to a dialysis system pumping an artificial CSF (142 mM NaCl, 3.9 mM KCl, 1.2 mM CaCl_2_, 1 mM MgCl_2_, 1.35 mM Na_2_HPO_4_, pH 7.4) at a flow rate of 1 *μ*L min^−1^. The probe protruded 1 mm from the tip of the guide shaft. Six hours after insertion of the probe (stabilization period), sampling was carried out. Six samples (20 *μ*L each) were collected every 20 min along 2 hours for each freely moving animal.

### 2.6. High-Performance Liquid Chromatography

Analysis of *γ*-aminobutyric acid (GABA) and glutamate (GLU) basal levels from microdialysates was performed using high performance liquid chromatography (HPLC) coupled to a fluorimetric detection system. A sample automatic derivatization (Waters 2690 Alliance) with *o*-phthalaldehyde was followed [11, 12, 19]. Resolution was obtained through a C18 reverse-phase chromatographic column coupled to the fluorometric detection (Waters 474; excitation wavelength 350 nm, emission wavelength recorder 450 nm). Buffer and gradient program was as follows: by definition, solvent A: 0.1 M sodium acetate pH 5.8/methanol 20/80; solvent B: 0.1 M sodium acetate pH 5.8/methanol 80/20; solvent C: 0.1 M sodium acetate pH 6.0/methanol 80/20. Concerning the gradient program, initial isocratic step 5% A, 95%C from 0 to 5 min; 15% A, 85% B from 4 to 5 min and then isocratic until 9 min; 22% A, 66% B until 14.5 min and then 34% A, 66% B until 17 min; 5% A, 95% C until 19 min and then isocratic until 23 min. Flow rate was 0.9 mL min^−1^. Homoserine was used as internal standard, and amino acid concentrations were calculated from a linear standard curve built upon known concentrations of injected amino acids. Area of the peaks was used to make comparisons (Waters Millenium 32).

### 2.7. Rearing Environments

Enriched environment consisted of a large cage (100 × 50 × 82 cm) with a wire mesh lid containing several food hoppers, a running wheel, and differently shaped objects (tunnels, shelters, stairs) that were repositioned once per day and completely substituted with others once per week, as described previously [12, 19]. Every enriched cage housed at least six rats. Standard conditions consisted of standard laboratory cages (40 × 30 × 20 cm) housing each 2 rats. Litter and food were the same in both experimental conditions; food and water provided *at libitum*.

### 2.8. Real-Time PCR

After respective treatments, Oc1b was dissected and immediately frozen. RNA purification was performed according to the standard Trizol procedure (Invitrogen). Purified RNA was treated with DNAse (Fermentas), and cDNA was synthesized from 1 ug of RNA (Invitrogen). Real-time PCR was carried out to determine relative enrichment in the samples using the SYBER Green method according to the manufacturer instructions (SYBR Green I master, Light cycler 480, Roche Diagnostics). The comparative Ct method was used to determine the normalized changes of the target gene relative to a calibrator reference, as described previously [15]. mRNA quantification samples were normalized to GAPDH levels. As calibrator reference we referred to Ct from control animal samples. Gene expression patterns of interest (see Table 1) were analyzed at two different time points during EE: after 2 and 7 days, respectively, during the last week of EE. Respective control animals housed under standard conditions were similarly treated.

## 3. Results

**Figure 1:**
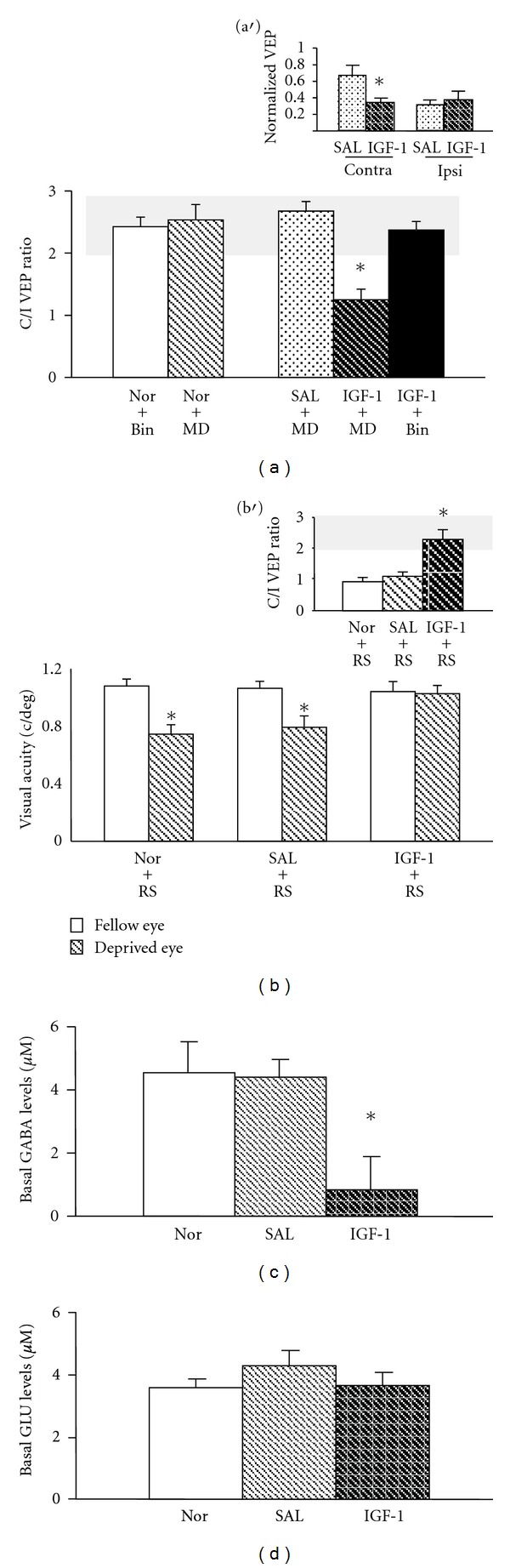
IGF-1 treatment reactivates plasticity in the adult visual cortex and decreases intracortical GABA levels. (a) An OD shift in favor of the open eye was evident in IGF-1-infused rats (IGF-1+MD; *n* = 5, C/I VEP ratio = 1.2 ± 0.17) with respect to adult animals with binocular vision (Nor+Bin; *n* = 5, C/I VEP ratio = 2.42 ± 0.16, one-way ANOVA *P* = 0.0002, post hoc Holm Sidak method, *P* < 0.05). No modification of the C/I VEP ratio was detected in either saline-treated animals (SAL+MD; *n* = 5, C/I VEP ratio = 2.67 ± 0.15, One-way ANOVA, post hoc Holm Sidak method) or normal adult rats (Nor+MD; *n* = 7, C/I VEP ratio = 2.53 ± 0.25, One-way ANOVA, post hoc Holm Sidak method). IGF-1 treated rats with binocular vision (IGF-1+Bin; *n* = 3) exhibited a C/I VEP ratio (2.37 ± 0.12) completely comparable to that of untreated Nor+Bin animals (One-way ANOVA, post hoc Holm Sidak method). Insert: the change of OD in IGF-1-treated rats was due to a decrease of the deprived eye (contra) strength. The amplitude of VEPs recorded in response to the stimulation of the occluded eye in IGF-1+MD rats (0.35 ± 0.05) was significantly lower with respect to that obtained in saline-treated animals following MD (0.68 ± 0.12; *t*-test; *P* = 0.047). No difference was detected in VEP amplitudes recorded after the stimulation of the open (Ipsi) eye (IGF-1+MD: 0.38 ± 0.11; SAL+MD: 0.32 ± 0.05; *t*-test; *P* = 0.627). VEP amplitudes at the recording site in the VC contralateral to the occlusion were normalized to the sum of the response to stimulation of the contralateral and ipsilateral eye, as described previously [10–12]. (b) Full rescue of VA was evident in reverse-sutured IGF-1 treated rats (IGF-1+RS, *n* = 5): VA of the long-term deprived eye (0.93 ± 0.06 cycles per degree, c/deg) was not different from that of the fellow eye (0.94 ± 0.04 c/deg; paired *t*-test, *P* = 0.837). No sign of recovery was detected either in reverse-sutured animals infused with saline (SAL+RS, *n* = 5; VA for the long-term deprived eye = 0.72 ± 0.03 c/deg; VA for the open eye 0.96 ± 0.01 c/deg; paired *t*-test, *P* < 0.001) or in reverse-sutured normal rats (Nor+RS, *n* = 5; VA for the long-term deprived eye = 0.67 ± 0.01 c/deg; VA for the open eye 1.03 ± 0.03 c/deg; paired *t*-test; *P* < 0.001) Insert, while in Nor-RS rats (C/I VEP ratio = 1.02 ± 0.08) there was no recovery of binocularity (One-way ANOVA, post hoc Holm Sidak method); in IGF1+RS the C/I VEP ratio is in the range of normal adult values (C/I VEP ratio 2.12 ± 0.13, One-way ANOVA, *F*_(3-16)_ = 40.24, *P* < 0.0001, post hoc Holm Sidak method). No recovery of OD was detected in SAL+RS animals (*n* = 5; C/I VEP ratio = 0.86 ± 0.11; One-way ANOVA, post hoc Holm Sidak method). (c) Extracellular GABA levels were significantly lower in the VC of IGF-1-treated rats (IGF-1; *n* = 6; 0.87 ± 0.17 *μ*M) with respect to saline-treated (SAL; *n* = 5; 4.46 ± 0.17 *μ*M) and untreated animals (Nor; *n* = 8,4.62 ± 0.97 *μ*M; One-way ANOVA, *F*_(2–16)_ = 7.445, *P* = 0.0052, post hoc Tukey test, *P* < 0.05). (d) No change in glutamate (GLU) levels between IGF-1-treated (3.88 ± 0.4 *μ*M) and control groups (SAL: 4.48 ± 0.6 *μ*M; Nor: 3.74 ± 0.3 *μ*M) was detected (One-way ANOVA, *F*_(2–15)_ = 0.744, *P* = 0.491). The grey box denotes the C/I VEP ratio range in adult normal animals. *Statistical significance. Error bars indicate SEM.

To address the functional relevance of IGF-1 signaling in the process of plasticity reactivation, we initially evaluated OD plasticity in adult rats that were infused in the VC with IGF-1 (0.5 *μ*g *μ*L^−1^), via osmotic minipumps, during 2 weeks and monocularly deprived during the last week of treatment. Plasticity was assessed by electrophysiological recordings of VEPs in the binocular region of the primary VC contralateral to the deprived eye (see methods). We measured the contralateral-to-ipsilateral (C/I) VEP ratio, which is the ratio of VEPs amplitudes recorded by stimulating the eye contralateral and ipsilateral, respectively, to the VC where recording is performed [29]. The C/I ratio was in the range of 2-3 in adult animals with binocular vision (C/I VEP ratio 2.42 ± 0.16; *n* = 5), reflecting the predominance of crossed fibers in the rat retinal projections. As shown in Figure 1(a), a significant shift of OD after MD was observed in IGF-1 treated rats (IGF-1+MD; C/I VEP ratio = 1.2 ± 0.17; *n* = 5, One-way ANOVA, *F*_(4–21)_ = 8.838, *P* = 0.0002, post hoc Tukey's test, *P* < 0.05) but not in rats infused with vehicle solution (SAL+MD; C/I VEP ratio = 2.67 ± 0.15, *n* = 5) or normal animals (Nor+MD; C/I VEP ratio = 2.53 ± 0.25; *n* = 7). No modification of OD was observed in control IGF-1 infused rats with binocular vision, indicating that IGF-1 treatment *per se* does not alter OD properties of VC neurons (IGF-1+Bin; C/I VEP ratio = 2.37 ± 0.14, *n* = 3).

To examine whether the OD shift observed in IGF-1+MD animals was due to a weakening of the deprived eye strength or to a strengthening of open eye responses [30, 31], we compared VEP amplitudes in response to the stimulation of each eye in IGF1- and saline-treated animals after MD. We report that the amplitude of VEPs recorded in response to stimulation of the occluded eye in IGF-1+MD rats (VEP amplitude 0.35 ± 0.05) was significantly lower (*t*-test; *P* = 0.047, *n* = 5) with respect to that obtained in saline-treated animals (VEP amplitude = 0.68 ± 0.12) after MD (Figure 1(a), Insert). No difference was detected in VEP amplitudes recorded after stimulation of the open eye (IGF-1+MD: VEP amplitude = 0.38 ± 0.11; SAL+MD: VEP amplitude = 0.32 ± 0.05).

We next assessed whether the potential for the reactivation of plasticity caused by IGF-1 treatment could be employed to promote the recovery of sensory functions from long-term deprivation using amblyopia as a paradigmatic model. Animals that were rendered amblyopic by long-term sensory deprivation were intracortically treated in adulthood with IGF-1 or vehicle solution, in parallel to reverse suture (RS), during two weeks (see methods). Visual acuity (VA) was measured by recordings of VEPs in the VC contralateral to the long-term deprived eye.  Figure 1(b) shows that VA of the long-term deprived eye (0.67 ± 0.01 cycles per degree, c/deg) was significantly lower (paired *t*-test; *P* = 0.0016, *n* = 5) than that of the fellow eye (0.97 ± 0.02 c/deg) in amblyopic animals after RS (Nor+RS). In contrast, a full rescue of VA was observed in reverse-sutured IGF-1 treated rats (IGF-1+RS; VA for the long-term deprived eye = 0.93 ± 0.06 c/deg; VA for the open eye 0.94 ± 0.04 c/deg; paired *t*-test; *P* = 0.229, *n* = 5). No sign of recovery was detected in control amblyopic animals infused with vehicle solution after RS (SAL+RS).

We also evaluated OD recovery by measuring the C/I VEP ratio in the same rats in which we measured VA (Figure 1(b), Insert). No recovery of binocularity was observed in adult amblyopic rats after RS (Nor+RS; C/I VEP ratio = 1.02 ± 0.08; *n* = 5). In contrast, the OD of visual cortical neurons was dominated by the contralateral eye in IGF1-treated animals (IGF-1+RS), showing a mean VEP ratio in the range of normal adult values and predictive of a full recovery of binocularity (C/I VEP ratio = 2.12 ± 0.13; *n* = 5, One-way ANOVA, *F*_(3–16)_ = 40.24, *P* < 0.0001, post hoc Tukey's test, *P* < 0.05). No rescue of OD was detected in control vehicle-treated rats (SAL+RS; C/I VEP ratio = 0.86 ± 0.11).

 Because there is evidence that a reduction of local inhibitory transmission underlies the reinstatement of plasticity in the adult visual system [9–12, 19], we investigated whether the IGF-1-mediated reactivation of plasticity was accompanied by a reduction of local GABAergic inhibition. *In vivo* brain microdialysis revealed that extracellular basal levels of GABA were markedly reduced (Figure 1(c); One-way ANOVA, *F*_(2–16)_ = 7.445, *P* = 0.0052, post hoc Tukey's test, *P* < 0.05) in the VC of IGF-1 treated rats (0.87 ± 0.2 *μ*M; *n* = 6) as compared to both normal rats (4.62 ± 0.97 *μ*M; *n* = 8) and vehicle-treated counterparts (4.46 ± 0.5 *μ*M; *n* = 5). No significant difference in basal glutamate (GLU) levels between any of the experimental groups was detected (Figure 1(d)).

**Figure 2:**
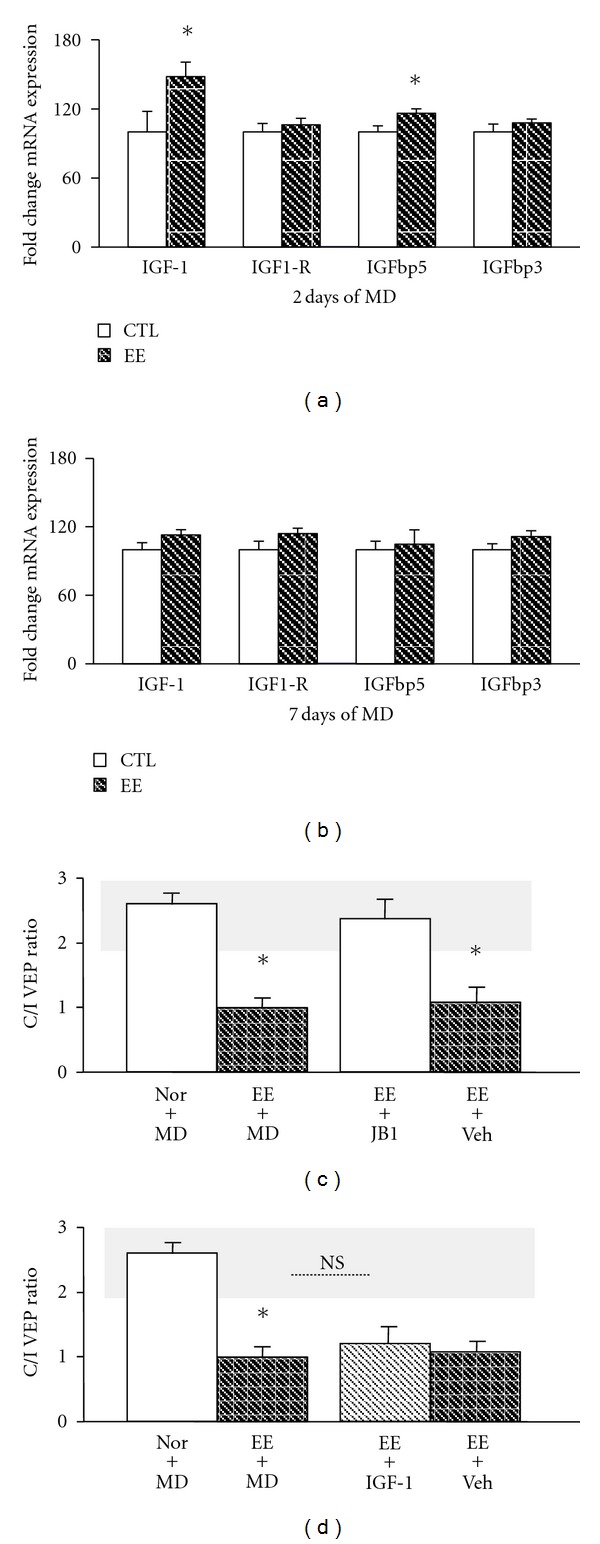
The upregulation of IGF-1 expression in EE animals mediates the effects of enriched experience on adult VC plasticity. (a) Analysis of gene expression by RT-PCR revealed that IGF-1 and IGFbp5 expression increased in the VC of EE animals after 2 days of MD with respect to SC animals similarly treated (*n* = 8 for both experimental groups;* t*-test; respectively, *P* = 0.034 and *P* = 0.030). In contrast, no modifications of either IGF1-R (*t*-test; *P* = 0.523) or IGFbp3 (*t*-test; *P* = 0.324) expression were detected. (b) The expression of IGF-1, IGF-1R, IGFbp5, and IGFbp3 in the VC was not different between EE animals monocularly deprived for 7 days and SC rats similarly treated. (c) JB1 infusion prevented the OD shift induced by MD in EE animals: no difference in C/I VEP ratio between normal animals (Nor+MD; *n* = 5, 2.67 ± 0.15) and EE rats treated with JB1 subjected to MD detected (EE+JB1; *n* = 6; 2.37 ± 0.09; One-way ANOVA, post hoc Holm Sidak method), while monocularly deprived EE animals (EE+MD; *n* = 6; 0.99 ± 0.07) and EE rats treated with vehicle (EE+Veh; *n* = 5; 1.08 ± 0.05) showed an OD shift in favor of the open eye (One-way ANOVA, *F*_(5–27)_ = 46.48, *P* < 0.0001, post hoc Holm Sidak method). (d) Coupling enriched experience with IGF-1 treatment (EE+IGF1 rats) did not further enhance the OD shift induced by MD in EE animals: the C/I VEP ratio measured in EE+IGF1 rats (*n* = 5; 1.08 ± 0.07) was completely comparable to that reported for EE animals (EE+MD), while it differed from that recorded in Nor+MD animals (One-way ANOVA *F*_(5–27)_ = 46.48, *P* < 0.0001, post hoc Holm Sidak method). The grey box denotes the C/I VEP ratio range in adult normal animals. *Statistical significance. NS: Not significant. Error bars indicate SEM.

 Since it has been demonstrated that IGF-1 is a crucial molecule underlying EE effects on visual system development [27], we next addressed whether IGF-1 mediates the EE-induced reactivation of plasticity in the adult VC. We used real-time PCR to investigate the expression of IGF-1 pathway genes in the VC of adult rats that were exposed to EE during 2 weeks. We assessed gene expression in two different groups of adult animals that were monocularly deprived for either 2 or 7 days, respectively, during the last week of EE (see methods). Interestingly, expression of IGF-1 significantly increased (*t*-test; *P* = 0.034,  *n* = 8) after 2 but not 7 days of MD in the VC of EE animals as compared to respective controls (Figures 2(a) and 2(b)). No change in the expression of the IGF-1 receptor was observed. Expression of the IGF-1 binding protein 5 (IGFbp5) increased after 2 days of MD (*t*-test; *P* = 0.030, *n* = 8), whereas no modification of IGFbp3 expression was evidenced in any of the experimental groups (Figures 2(a) and 2(b)).

 We next examined whether IGF-1 is causally linked to the EE-induced process of plasticity reactivation. Adult rats were intracortically infused with the IGF-1 receptor antagonist JB1 (10 *μ*g mL^−1^) in parallel to the period of EE. This treatment has been previously used to assess development of normal visual functions in rodents [27]. The C/I VEP ratio shifted in response to MD (One-way ANOVA, *F*_(5–27)_ = 46.48, *P* = 0.0001, post hoc Tukey's test, *P* < 0.05) in adult animals exposed to EE (EE+MD; C/I VEP ratio 0.99 ± 0.07, *n* = 6) but not in EE rats intracortically infused with JB1 (EE+JB1; C/I VEP ratio 2.37 ± 0.09, *n* = 6), indicating that IGF-1 signaling is critical for the effects caused by EE in VC plasticity (Figure 2(c)). EE animals infused with vehicle solution (EE+Veh), instead, showed a marked shift of OD after MD.

We finally examined whether the physiological reduction of inhibition that occurs under conditions of EE [12, 19] occludes phenomena of plasticity caused by intracortical IGF-1 administration. Interestingly, the shift of OD induced by EE (EE+MD; C/I VEP ratio 0.99 ± 0.07) did not differ from that caused by EE+IGF-1 treatment (C/I VEP ratio 1.08 ± 0.07). These findings suggest that the reduction of inhibition is, at least, one of the physiological mechanisms by which IGF-1 restores plasticity in the adult VC (Figure 2(d)).

## 4. Discussion

Our findings demonstrate that intracortical IGF-1 treatment effectively reinstates neural plasticity in the adult visual system, as indicated by the enhanced susceptibility of VC neurons in response to MD in adult life. We provide evidence that OD plasticity in IGF-1 animals occurs through a juvenile-like mechanism: the OD shift observed in IGF1-treated animals, indeed, was entirely due to a marked reduction in the deprived eye response, an event detected in the developing VC. Even if a link between brain plasticity and IGF-1 has been suggested by previous findings showing that IGF-1 increases hippocampal neurogenesis [22] and mediates the enhancement of synaptic plasticity induced by physical activity in the adult brain [21–24], this is the first time that the IGF-1 effects have been studied using the highly reliable paradigm of OD plasticity.

The possibility to enhance plasticity in the adult nervous system through IGF-1 infusion may be used to promote the recovery of sensory functions after long-term deprivation. Accordingly, we found that exogenous IGF-1 administration is effective in the treatment of amblyopia, a pathological condition that lacks of a suitable treatment in adulthood.

Recent studies indicate that a great number of experimental strategies promoting juvenile-like plasticity in the adult brain cause a shift of the intracortical excitatory-inhibitory balance [9–15, 19]. Consistently, the effects caused by IGF-1 in VC plasticity were accompanied by a decrease of extracellular GABA levels. This suggests that a downregulation of the inhibitory tone may be, at least, one of the mechanisms underlying the IGF-1-induced reactivation of plasticity. Since IGF-1 enhances neuronal glucose uptake [32], an increase of glucose metabolism may also contribute to the IGF-1-induced process of plasticity reactivation. In addition, IGF-1 may act as a subset of neurotrophic factors. IGF-1 exogenous administration in parallel to MD during the CP, indeed, recalls the effects caused by the neurotrophins NGF [33] and NT-4 [34] in the developing visual system. In this context, it is intriguing that anti-NGF antibodies, a treatment previously shown to influence VC plasticity [33] and to prolong the CP for plasticity in the visual system [35], determine a major remodeling of the inhibitory-excitatory equilibrium in the hippocampus of 6 months old anti-NGF AD11 transgenic mice with a shift of GABA activity from hyperpolarizing to depolarizing, due to a downregulation of the KCC2 chloride transporter [36].

**Figure 3:**
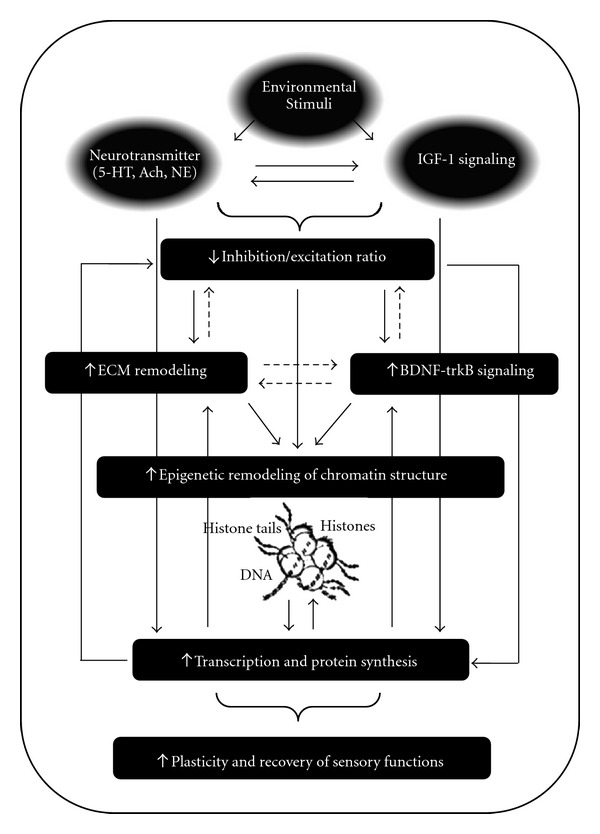
The process of plasticity reactivation induced by EE is associated with signal transduction pathways that involve the activation of long-distance neuromodulatory systems and IGF-1 signaling. We propose a model in which the interplay between 5-HT and IGF-1 transmission, in parallel or in series, shifts the inhibitory/excitatory balance in favour of excitation thus activating intracellular mechanisms that eventually promote epigenetic modifications of chromatin structure that, in turn, allow for the expression of plasticity genes in adult life. A pharmacological reduction of inhibitory transmission could promote Bdnf expression and activate physiological mechanisms that may drive the degradation of extracellular matrix (ECM) components that are inhibitory for plasticity. 5-HT and IGF-1 signaling, respectively, may also directly activate Bdnf expression or enhance the ECM remodeling. Bdnf-trkB signaling might upregulate additional gene expression patterns associated with functional modifications in the VC. This could also alter the balance of intracortical inhibition and excitation. Degradation of ECM components may modify the inhibition/excitation ratio in the visual system. The interaction between BDNF-trkB signaling and ECM reorganization has yet to be explored. Continuous arrows represent established interactions between molecular and cellular processes mentioned (boxes). Dashed lines represent interactions that remain to be ascertained.

We observed that the reinstatement of plasticity caused by EE was paralleled by a transient enhancement of IGF-1 expression and that antagonizing IGF-1 signaling in EE animals counteracted the process of plasticity reactivation. Previous studies have demonstrated that the restoration of plasticity is a multifactorial event that comprises the action of different cellular and molecular mechanisms, working in parallel or in series, the sum of which results in the activation of intracellular signal transduction pathways regulating the expression of plasticity genes in the adult brain [15, 37, 38], reviewed in [17, 20, 39]. The observation that serotonin (5-HT) triggers a transient epigenetic mechanism that reinstates OD plasticity in adulthood [15] and mediates the plastic outcome of EE in the adult visual system [12] may suggest that 5-HT sets in motion physiological mechanisms underlying these plastic phenomena. However, our present data brings IGF-1 as an additional player into this context. A role for a combined action of 5-HT and IGF-1 signaling as mediators of adult plasticity may be exemplified by a model in which the 5-HT transmission increases IGF-1 expression, which in turn could be, at least, one of the mechanisms by which the reduction of local GABAergic inhibition and enhancement of BDNF levels are accomplished under EE conditions (Figure 3). Consistently, a pharmacological increase of 5-HT transmission promotes IGF-1 expression in different areas of the adult brain [40, 41], whereas IGF-1 signaling controls BDNF expression [42]. This is also in line with the observation that EE upregulates IGF-1 signaling in the adult rat hippocampus and sensory-motor cortex [43].

Another possibility is that IGF-1 enhances 5-HT transmission, setting in motion downstream mechanisms that restore plasticity in adult life: hippocampal IGF-1 administration, for instance, has been reported to initiate a long-lasting cascade of neurochemical effects involving increased serotonin levels [44]. We cannot rule out that serotoninergic transmission and IGF-1 signaling act as independent pathways converging, in parallel, on inhibitory transmission and BDNF signaling. Because the largest source of IGF-1 in the adult brain comes from the periphery [21–24], our findings do not exclude the possibility that circulating IGF-1 levels may also contribute to the effects caused by EE in VC plasticity.

It is also worth mentioning that IGFbp5 expression increased in the VC of EE animals after 2 days of MD. This is consistent with previous findings showing that MD up regulates IGFbp5 during the CP at the level of mRNA and protein [25] and provides further support for an increased bioavailability of local IGF-1 in the visual system of adult animals after brief exposure to EE conditions [45]. Moreover, there is evidence that IGFbp5 increases the conversion of plasminogen into the proteolytic enzyme plasmin [46], which suggests that the associated cleavage of extracellular matrix proteins is likely to contribute to the plastic outcome of EE in the adult. The process of plasticity reactivation caused by EE, indeed, is associated to a reduction of extracellular matrix molecules that are repressive for plasticity [19]. In line with this, a pharmacological removal of extracellular matrix components restores VC plasticity in adulthood [47, 48].

## 5. Conclusion

We found that IGF-1 is a molecular factor that reinstates juvenile-like plasticity in adult VC circuitries and that this plastic phenomenon is likely to be mediated by a reduction of inhibitory transmission. Moreover, IGF-1 emerges as a critical player in the process of plasticity reactivation caused by EE in adulthood.

Given the effectiveness of IGF-1 in restoring plasticity in the adult VC, one may speculate that the beneficial effects exerted by this treatment could be exploited for clinical application. Since deterioration in functional plasticity contributes to the pathogenesis of several brain diseases, IGF-1 arises as a therapeutic strategy to delay the progression and/or to ameliorate the symptoms of neurodegenerative disorders such as Alzheimer's disease. This notion is supported by the recent observation that exogenous administration of IGF-2 promotes memory consolidation and retention in rodents [49].

Human studies have shown a positive correlation between IGF-1 and mental abilities [21, 50–54], and IGF-1 has been employed for the treatment of diabetes [55, 56], growth failure [57, 58], and motor neuronal disorders [59, 60]. Together with our finding that IGF-1 promotes plasticity in the adult nervous system, these observations suggest that systemic IGF-1 delivery could be used to enhance plasticity as a strategy for brain repair in adult life. This may be of relevance in neurological disorders in which synaptic plasticity is compromised because of excessive intracortical inhibition [61–63].

## Figures and Tables

**Table 1:** Detailed description of the oligos (sense/antisense) used to amplify genes of interest.

Gene	Oligo sense	Oligo antisense
IGF-1	CAGTTCGTGTGTGGACCAAG	CAACACTCATCCACAATGCC
IGF1-R	TGAACCCCGAGTATTTCAGC	GGCCACTCCTTCATAGACCA
IGFbp5	GGTTTGCCTCAACGAAAAGA	GAAGACCTTCGGGGAGTAGG
IGFbp3	GCTATGACACCAAGGGGAA	TTGTTGGCAGTCTTTTGTGC
